# Supplementary material for: Content-rich biological network constructed by mining PubMed abstracts
Source: BMC Bioinformatics. 2004 Oct 8;5:147. doi: 10.1186/1471-2105-5-147 (PMC528731; doi:10.1186/1471-2105-5-147)
Supplement: Additional File 2 — The original results of the above study (non-essential files are deleted to keep the file size under the limit set by BMC bioinformatics). [file 1471-2105-5-147-S2.bz2 › chilibotAdditionalFile2/dip30/392ID8700872E9878/html/FLT1_PGF.html]

 


 **FLT1** and **PGF** 
  
Found 101 abstracts in PubMed, retrieved 30.  
 

 What does Google say? 
 PDF only 
| .edu only 

---

**Interactive relationship** (e.g. stimulation, inhibition, etc)

**Neutral relationship**- Placental growth factor  [ **PGF** ]  reconstitutes hematopoiesis by recruiting VEGFR1  [ **FLT1** ]  stem cells from bone marrow microenvironment.  Ref: 12091880 Nat Med, 2002
- Thus, PlGF  [ **PGF** ]  promotes recruitment of VEGFR1  [ **FLT1** ]  HSCs from a quiescent to a proliferative BM microenvironment, favoring differentiation, mobilization and reconstitution of hematopoiesis.  Ref: 12091880 Nat Med, 2002
- PlGF  [ **PGF** ]  promotes recruitment of VEGFR1  [ **FLT1** ]  HSCs from a quiescent to a proliferative microenvironment within the BM, favoring differentiation, mobilization, and reconstitution of hematopoiesis.  Ref: 12799282 Ann N Y Acad Sci< MedlineTA>Ann N Y Acad Sci, 2002
- Similarly, VEGF and KDR selective mutant induced more potent vasorelaxation than Flt  [ **FLT1** ]  selective mutant or placenta growth factor  [ **PGF** ]  that binds Flt 1  [ **FLT1** ]  only P< ; 0.01, and the vasorelaxation to KDR selective mutant was not significantly different at low concentrations but less than that to VEGF at high concentrations.  Ref: 12052848 Hypertension, 2002
- Furthermore, the VEGF  **PGF**  heterodimer activated intramolecular VEGF receptor cross talk through formation of Flk1  **Flt1**  heterodimers.  Ref: 12796773 Nat Med< MedlineTA>Nat Med, 2002
- We show that while a significant change in the formation of endothelial surface growth factor VEGFR1  [ **FLT1** ]  complexes is predicted in the presence of PlGF  [ **PGF** ] , the number of VEGFR2 containing signaling complexes is relatively unchanged.  Ref: 12714333 Am J Physiol Heart Circ Physiol< MedlineTA>Am J Physiol Heart Circ Physiol, 2002
- Here we report that placental growth factor  [ **PGF** ]   **PGF** , also known as PlGF  [ **PGF** ]  regulates inter and intramolecular cross talk between the VEGF RTKs  **Flt1**  and Flk1.  Ref: 12796773 Nat Med< MedlineTA>Nat Med, 2002
- To understand the molecular basis of the biological activity of VEGF E, we have functionally mapped residues important for interaction of VEGF E with VEGFR  [ **FLT1** ]  2 by exchanging the domains between VEGF E NZ 7 and PlGF  [ **PGF** ] , which binds only to VEGFR 1  [ **FLT1** ]  Flt 1  [ **FLT1** ] .  Ref: 12551914 J Biol Chem, 2003
- Therefore, we used the VEGF homologue placenta growth factor  [ **PGF** ]  PlGF  [ **PGF** ] , which only binds to VEGFR 1  [ **FLT1** ]  and VEGF E, which only recognizes VEGFR  [ **FLT1** ]  2.  Ref: 12600898 Circ Res, 2003
- Mice expressing a truncated form of the specific receptor for PlGF  [ **PGF** ] , the VEGF receptor 1 VEGFR 1  [ **FLT1** ] , show impaired angiogenesis similar to that observed in Plgf  [ **PGF** ]  mice.  Ref: 12242046 Trends Cardiovasc Med, 2002
- Vessels of PlGF  [ **PGF** ]  implants were lined by vWF cells, which were mostly flt 1  [ **FLT1** ] .  Ref: 12673673 Int J Cancer, 2003
- Placental Growth Factor  [ **PGF** ]  PlGF  [ **PGF** ]  competes with Vascular Endothelial Growth Factor VEGF for binding to VEGF Receptor 1 VEGFR1  [ **FLT1** ] , but does not bind VEGFR2.  Ref: 12714333 Am J Physiol Heart Circ Physiol< MedlineTA>Am J Physiol Heart Circ Physiol, 2003
- Placental growth factor  [ **PGF** ]  PlGF  [ **PGF** ]  and vascular endothelial growth factor VEGF are involved in placental angiogenesis through interactions with the VEGFR 1  [ **FLT1** ]  and VEGFR  [ **FLT1** ]  2 receptors.  Ref: 12740423 J Physiol< MedlineTA>J Physiol, 2003
- In this review, we describe the molecular properties of PlGF  [ **PGF** ]  and VEGFR 1  [ **FLT1** ]  and how this translates into an important role for PlGF  [ **PGF** ]  in the angiogenic switch in pathological angiogenesis, by interacting with VEGFR 1  [ **FLT1** ]  and synergizing with VEGF.  Ref: 12871269 J Thromb Haemost, 2003
- They belong to the larger family which also includes VEGF, placenta growth factor  [ **PGF** ]  PlGF  [ **PGF** ]  and VEGF B, VEGF C and VEGF D are ligands for the endothelial cell specific tyrosine kinase receptors VEGFR  [ **FLT1** ]  2 and VEGFR  [ **FLT1** ]  3.  Ref: 12888864 Thromb Haemost, 2003
- Placental growth factor  [ **PGF** ]  1 PlGF  [ **PGF** ]  1, which exclusively binds VEGFR 1  [ **FLT1** ] , decreased hyperoxia induced retinal vaso obliteration from 22.2% to 5.1%.  Ref: 12840058 J Clin Invest, 2003
- SU5416 inhibited cell migration of human vascular endothelial cells expressing both Flt 1  [ **FLT1** ]  and KDR in response to VEGF and also inhibited the cell migration in response to placenta growth factor  [ **PGF** ]  PIGF, a specific ligand for Flt 1  [ **FLT1** ] .  Ref: 12489845 Mol Cancer Ther, 2002
- Even though VEGF and  **PGF**  both bind  **Flt1** ,  **PGF**  uniquely stimulated the phosphorylation of specific  **Flt1**  tyrosine residues and the expression of distinct downstream target genes.  Ref: 12796773 Nat Med< MedlineTA>Nat Med, 2002
- PlGF  [ **PGF** ]  enhanced early phases of BM recovery directly through rapid chemotaxis of VEGFR1  [ **FLT1** ]  BM repopulating and progenitor cells.  Ref: 12091880 Nat Med, 2002
- Furthermore, the results indicated that VEGF and PlGF  [ **PGF** ]  stimulate inflammatory cell migration by autocrine regulation via the Flt 1  [ **FLT1** ]  receptor in the CAM placenta.  Ref: 12454810 Hum Pathol, 2002

**Non-interactive relationship** (e.g. studied together, co-existance, homology, etc.)

- Expression of vascular endothelial growth factor, placental growth factor  [ **PGF** ] , and their receptors Flt 1  [ **FLT1** ]  and KDR in human placenta under pathologic conditions.  Ref: 12454810 Hum Pathol, 2002
- Role of PlGF  [ **PGF** ]  in the intra and intermolecular cross talk between the VEGF receptors  **Flt1**  and Flk1.  Ref: 12796773 Nat Med< MedlineTA>Nat Med, 2002
- METHODS AND RESULTS Exposure to hypoxia induced a potentiation of cultured cell proliferation in response to either the agonist for the VEGF receptor 1 flt 1  [ **FLT1** ]  placental growth factor  [ **PGF** ]  PlGF  [ **PGF** ]  1 or to VEGF A.  Ref: 12062723 Cardiovasc Res, 2002
- We have investigated the expression patterns of VEGF, placental growth factor  [ **PGF** ]  PlGF  [ **PGF** ] , and their receptors fms like tyrosine kinase Flt 1  [ **FLT1** ]  and kinase insert domain containing region KDR in placentas with histopathological changes.  Ref: 12454810 Hum Pathol, 2002
- Thus, PlGF  [ **PGF** ]  and  **Flt1**  constitute potential candidates for therapeutic modulation of angiogenesis and inflammation.  Ref: 12091877 Nat Med, 2002
- Placental growth factor  [ **PGF** ]  PlGF  [ **PGF** ] , which signals through VEGFR1  [ **FLT1** ] , restored early and late phases of hematopoiesis following BM suppression.  Ref: 12091880 Nat Med, 2002
- Revascularization of ischemic tissues by PlGF  [ **PGF** ]  treatment, and inhibition of tumor angiogenesis, arthritis and atherosclerosis by anti  **Flt1** .  Ref: 12091877 Nat Med, 2002
- Positive immunostaining for VEGF, PlGF  [ **PGF** ] , and Flt 1  [ **FLT1** ]  was observed in infiltrated neutrophils and macrophages in the placentas with chorioamnionitis CAM.  Ref: 12454810 Hum Pathol, 2002
- activation of VEGFR 1  [ **FLT1** ]  by PlGF  [ **PGF** ]  1 is a selective strategy for preventing oxygen induced retinal ischemia without provoking retinal neovascularization.  Ref: 12840058 J Clin Invest, 2003
- Together, these data indicate that the arteriogenic activity observed with the VEGFR 1  [ **FLT1** ]  specific PlGF  [ **PGF** ]  is caused by its monocyte activating properties.  Ref: 12600898 Circ Res, 2003
- Placental growth factor  [ **PGF** ]  and its receptor, vascular endothelial growth factor receptor 1  [ **FLT1** ]  novel targets for stimulation of ischemic tissue revascularization and inhibition of angiogenic and inflammatory disorders.  Ref: 12871269 J Thromb Haemost, 2003
- Activation of  **Flt1**  by  **PGF**  resulted in intermolecular transphosphorylation of Flk1, thereby amplifying VEGF driven angiogenesis through Flk1.  Ref: 12796773 Nat Med< MedlineTA>Nat Med, 2003
- These data suggest a pivotal role for PlGF  [ **PGF** ]  and VEGFR 1  [ **FLT1** ]  in regulating VEGF A dependent angiogenesis under pathological conditions.  Ref: 12242046 Trends Cardiovasc Med, 2002
- Placental growth factor  [ **PGF** ]  PlGF  [ **PGF** ]  and its receptor Flt 1  [ **FLT1** ]  VEGFR 1  [ **FLT1** ]  novel therapeutic targets for angiogenic disorders.  Ref: 12543719 Ann N Y Acad Sci, 2002
- Synergistic effects observed in that experiment thus appear not to be due to VEGF displacement, but to a shift from VEGF VEGFR1  [ **FLT1** ]  to PlGF  [ **PGF** ]  VEGFR1  [ **FLT1** ]  complexes and an increase in total VEGFR1  [ **FLT1** ]  complexes.  Ref: 12714333 Am J Physiol Heart Circ Physiol< MedlineTA>Am J Physiol Heart Circ Physiol, 2002
- Here, we confirm that placental soluble fms like tyrosine kinase 1  [ **FLT1** ]  sFlt1, an antagonist of VEGF and placental growth factor  [ **PGF** ]  PlGF  [ **PGF** ] , is upregulated in preeclampsia, leading to increased systemic levels of sFlt1 that fall after delivery.  Ref: 12618519 J Clin Invest, 2003
- Tumors removed at 1 week intervals up to week 6 were probed by immunohistochemistry n = 3 11 samples for vascular endothelial growth factor VEGF, placental growth factor  [ **PGF** ]  PlGF  [ **PGF** ] , flk 1 and flt 1  [ **FLT1** ] , angiopoietin 1 and 2, and Tie 1 and 2.  Ref: 11948135 Clin Cancer Res, 2002
- The analysis of vascular endothelial growth factor receptor 1  [ **FLT1** ]  flt 1  [ **FLT1** ]  and vascular endothelial growth factor receptor 2 flk 1 indicates that the two receptors are induced in the skin endothelium of transgenic mice suggesting that both are involved in mediating the effect of overexpressed PlGF  [ **PGF** ] .  Ref: 12045226 J Cell Sci, 2002
- Similarly, PlGF  [ **PGF** ]  1 VEGF fails to activate the VEGFR  [ **FLT1** ]  2 mediated signaling pathways.  Ref: 12086892 Cancer Cell, 2002
- The mechanism whereby PlGF  [ **PGF** ]  enhanced early phases of BM recovery was mediated directly through rapid chemotaxis of readily available VEGFR1  [ **FLT1** ]  BM repopulating and progenitor cells.  Ref: 12799282 Ann N Y Acad Sci< MedlineTA>Ann N Y Acad Sci, 2002
- and displacement of VEGF from VEGFR1  [ **FLT1** ]  to VEGFR2 by PlGF  [ **PGF** ] , causing increased signaling through VEGFR2.  Ref: 12714333 Am J Physiol Heart Circ Physiol< MedlineTA>Am J Physiol Heart Circ Physiol, 2002
- The therapeutic potential of placental growth factor  [ **PGF** ]  PlGF  [ **PGF** ]  and its receptor  **Flt1**  in angiogenesis is poorly understood.  Ref: 12091877 Nat Med, 2002
- Efforts to therapeutically stimulate or inhibit vessel growth have been primarily focused on vascular endothelial growth factor VEGF and its receptor VEGFR  [ **FLT1** ]  2 Flk 1, while little attention has been devoted to the therapeutic potential for angiogenic disorders of placental growth factor  [ **PGF** ]  PlGF  [ **PGF** ] , a VEGF family member, and its receptor VEGFR 1  [ **FLT1** ]  Flt 1  [ **FLT1** ] .  Ref: 12543719 Ann N Y Acad Sci, 2002
- To dissect the signals of these two receptors, we generated transgenic mice overexpressing either the VEGFR  [ **FLT1** ]  2 specific ligand VEGF E NZ 7 or VEGFR 1  [ **FLT1** ]  specific ligand PlGF  [ **PGF** ]  II under the control of the Keratin 14 promoter.  Ref: 12565870 Biochem Biophys Res Commun, 2003
- Plasma elevation of placental growth factor  [ **PGF** ]  PlGF  [ **PGF** ] , which signals through VEGFR1  [ **FLT1** ] , but not VEGFR2, restored hematopoiesis during the early and late phases following BM suppression.  Ref: 12799282 Ann N Y Acad Sci< MedlineTA>Ann N Y Acad Sci, 2003
- This review discusses the possibility of using the PlGF  [ **PGF** ]  VEGFR 1  [ **FLT1** ]  pathway as an alternative target for angiogenic therapy.  Ref: 12242046 Trends Cardiovasc Med, 2002
- In contrast to VEGF and its receptor VEGFR  [ **FLT1** ]  2, PlGF  [ **PGF** ]  and its receptor VEGFR 1  [ **FLT1** ]  have been largely neglected and therefore their potential for therapy has not been previously explored.  Ref: 12871269 J Thromb Haemost, 2003
- VEGFR 1  [ **FLT1** ]  selective VEGF homologue PlGF  [ **PGF** ]  is arteriogenic evidence for a monocyte mediated mechanism.  Ref: 12600898 Circ Res, 2003
- Evaluation of collateral growth by determining collateral conductance and angiographic scores demonstrated that the VEGFR 1  [ **FLT1** ]  specific PlGF  [ **PGF** ]  contributed significantly more to arteriogenesis than the VEGFR  [ **FLT1** ]  2 specific VEGF E.  Ref: 12600898 Circ Res, 2003
- ... the expression of PlGF  [ **PGF** ]  and Flt 1  [ **FLT1** ]  is enhanced in villous trophoblasts by autocrine regulation.  Ref: 12454810 Hum Pathol, 2002
- When we focus on the VEGF receptors, recent findings suggest a role of VEGFR 1  [ **FLT1** ]  as a functional receptor for placenta growth factor  [ **PGF** ]  PlGF  [ **PGF** ]  and vascular endothelial growth factor A VEGF A in pericytes and vascular smooth muscle cells in vivo rather than in endothelial cells, and strongly suggest involvement of pericytes in early phases of angiogenesis.  Ref: 12597922 Prog Retin Eye Res, 2003
- Thus, PlGF  [ **PGF** ]  and Flt 1  [ **FLT1** ]  constitute potential candidates for therapeutic modulation of angiogenesis and inflammation.  Ref: 12543719 Ann N Y Acad Sci, 2002
- Thus, these preclinical studies show proof of principle that PlGF  [ **PGF** ]  and VEGFR 1  [ **FLT1** ]  are promising therapeutic targets to treat angiogenesis and inflammation related disorders.  Ref: 12871269 J Thromb Haemost, 2003
- This synergy has been hypothesized to be due to a combination of signaling by PlGF  [ **PGF** ]  through VEGFR1  [ **FLT1** ] .  Ref: 12714333 Am J Physiol Heart Circ Physiol< MedlineTA>Am J Physiol Heart Circ Physiol, 2003
